# Sphingosine 1-Phosphate (S1P) Signaling in Glioblastoma Multiforme—A Systematic Review

**DOI:** 10.3390/ijms18112448

**Published:** 2017-11-17

**Authors:** Shailaja Mahajan-Thakur, Sandra Bien-Möller, Sascha Marx, Henry Schroeder, Bernhard H. Rauch

**Affiliations:** 1 Department of Pharmacology, University Medicine Greifswald, 17487 Greifswald, Germany; mahajan.shailaja@gmail.com (S.M.-T.); sbien@uni-greifswald.de (S.B.-M.); 2 Clinic of Neurosurgery, University Medicine Greifswald, 17487 Greifswald, Germany; marxs@uni-greifswald.de (S.M.); henry.schroeder@uni-greifswald.de (H.S.)

**Keywords:** glioblastoma multiforme, Sphingosin-1-phosphate, S1P receptor signaling

## Abstract

The multifunctional sphingosine-1-phosphate (S1P) is a lipid signaling molecule and central regulator in the development of several cancer types. In recent years, intriguing information has become available regarding the role of S1P in the progression of Glioblastoma multiforme (GBM), the most aggressive and common brain tumor in adults. S1P modulates numerous cellular processes in GBM, such as oncogenesis, proliferation and survival, invasion, migration, metastasis and stem cell behavior. These processes are regulated via a family of five G-protein-coupled S1P receptors (S1PR1-5) and may involve mainly unknown intracellular targets. Distinct expression patterns and multiple intracellular signaling pathways of each S1PR subtype enable S1P to exert its pleiotropic cellular actions. Several studies have demonstrated alterations in S1P levels, the involvement of S1PRs and S1P metabolizing enzymes in GBM pathophysiology. While the tumorigenic actions of S1P involve the activation of several kinases and transcription factors, the specific G-protein (Gi, Gq, and G12/13)-coupled signaling pathways and downstream mediated effects in GBM remain to be elucidated in detail. This review summarizes the recent findings concerning the role of S1P and its receptors in GBM. We further highlight the current insights into the signaling pathways considered fundamental for regulating the cellular processes in GMB and ultimately patient prognosis.

## 1. Introduction

The lipid mediator sphingosine 1-phosphate (S1P) regulates a variety of cellular processes including inflammation, oncogenesis, metastasis, survival, stem cell behavior and the formation of microvascular networks, which provides nourishment to cancerous cells [1]. As a consequence, S1P has been increasingly recognized in recent years as an important oncogenic factor involved in numerous cancer categories including breast, colorectal, kidney, glioblastoma multiforme (GBM), lung, melanoma, and ovarian [2]. The S1P-related key players involved in the progression of such tumor types are S1P receptors (S1PRs), its metabolizing enzymes sphingosine kinases (SphK1 and SphK2), phosphatases, and the S1P lyase [2]. Intriguingly, intracellular secondary messenger functions of S1P and the consecutively activated signaling pathways are also considered pivotal in regulating the cellular processes during cancer pathology. Despite several studies showing the biological functions of S1P, it seems to be challenging to define the specific mechanism(s) involved in a particular cancer type. This most likely is owed to the complex nature of the S1P signaling system as S1P varies in cellular origin, cell type-specific actions, abundance or deficiency of the specific S1P receptors, and intracellular environment [3]. 

Over the last two decades or so, a large amount of information has become available regarding the role of S1P-mediated cellular processes in cancer development. Several studies have associated alteration in S1P levels, and the involvement of its receptors and metabolite enzymes in many types of cancer pathophysiology. In an animal model of intestinal tumorigenesis, increased S1P levels associated with reduced S1P lyase (SPL) expression and enzyme activity were observed compared to other local tissues [4]. In human ovarian cancer patients, S1P was elevated in the plasma and malignant ascites [5]. Another study described highly elevated S1P levels in a similar group of cancer patients as compared to controls [6]. High expressions of S1PR1, S1PR3, and the S1P generating enzyme SphK1 have been reported in breast cancer patients [7]. Overexpression of SphK1 has also been seen in both animal and xenograft models of several tumor types including a rat colon adenocarcinomas and a mouse leukemia model, and human breast, lung, and colon tumors, as compared to matched normal tissues [8]. An enhanced proliferation and decreased apoptosis correlated with high SphK1 expression in mouse breast cancer cells [9]. Elevated levels of S1P were also found in GBM when compared to non-malignant brain tissue. Poor prognosis of patients with GBM has recently been correlated with elevated expression SphK1 and S1PR2, whereas a high expression of S1PR1 resulted in an improved prognosis [10,11]. In this review, we exclusively focus on the literature that provides insights into the role of S1P signaling in the pathogenesis of GBM. 

## 2. Biosynthesis of Ceramide and Sphingolipids

**Figure 1:**
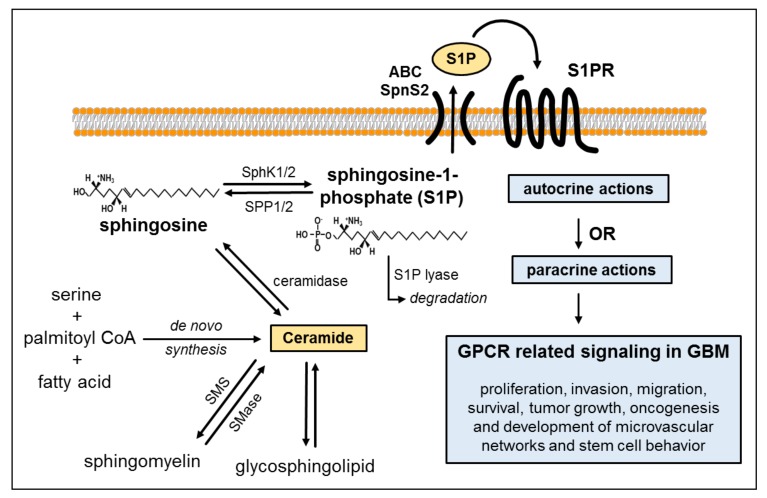
Biosynthesis of ceramide and sphingosine-1-phosphate (S1P) production. Ceramide *de novo* synthesis typically originates from the condensation of serine, palmitoyl-CoA and fatty acids—a multistep enzyme-catalyzed process. Ceramide can be transformed reversibly (indicated by the two-way arrows) into sphingomyelin by sphingomyelinase or to glycosphingolipids. It is further metabolized to sphingosine by ceramidase. Sphingosine can then be phosphorylated into S1P by the sphingosine kinase isoforms 1 and 2 (SphK1/2). This phosphorylation can be reverted by the S1P phosphatases 1 and 2 (SPP1/2), or irreversible degradation by S1P lyase can occur. S1P produced intracellularly is exported out of the cell via ATP-binding cassette transporters (ABC) transporters or spinster homolog 2 (Spns2), dependent on the cell type. Subsequently, it can act either in an autocrine or paracrine manner by binding to one of its receptors (S1PR1–5) to regulate multifaceted cellular functions via G-protein-mediated signaling. S1P promotes key processes of glioblastoma multiforme (GBM) pathogenesis which involve cell proliferation, invasion, migration, survival, tumor growth, oncogenesis and development of microvascular networks. Additional abbreviations used in the figure are defined as follows: SMase, sphingomyelinase; SMS, sphingomyelin synthase; S1PRs, S1P receptor(s).

Sphingolipid and particularly S1P synthesis are tightly controlled by the metabolism of ceramide. Ceramide biosynthesis occurs either *de novo* from serine, palmitoyl-CoA and fatty acids or from the breakdown of membrane-resident sphingomyelin [12]. Ceramide is converted to sphingosine by the enzymatic action of ceramidase. Ultimately, the bioactive lysophospholipid S1P is generated by phosphorylation of sphingosine—a reaction catalyzed by the isoenzymes SphK1 and SphK2. Sustaining a balance between S1P generation and degradation is critical for the regulation of cell growth and plays a key role in pathological processes such as carcinogenesis [13]. S1P degradation is achieved via reversible dephosphorylation by two S1P-specific phosphatases (SGPP1 and SGPP2) or via irreversible hydrolysis by S1P lyase (SGPL). S1P exerts actions either by binding to its specific receptors in autocrine or paracrine manner or through intracellular targets [14] (Figure 1). *In vitro* and *in silico* analyses showed that sphingosine metabolized in lysosomes was preferentially utilized for the generation of ceramide in astrocytes. In contrast, in glioma cells, sphingosine was primarily used for the production of survival-promoting S1P [15].

## 3. S1P Signaling in GBM

GBM is known as one of the most aggressively infiltrative primary brain tumors with dismal adult patient prognosis. According to the GBM pathogenesis, there are two subtypes distinguished—the primary glioblastoma, which develops *de novo* from glial progenitor cells, and the secondary glioblastoma, which arises from lower malignant astrocytic tumors. For both subtypes, many mutations have been described and both are highly infiltrating tumors [16]. Because of their highly invasive and proliferative nature, these tumors are frequently difficult to resect surgically. Unfortunately, despite surgical removal of the tumor and a multi-modal therapy (combined radio- and chemotherapy), the median survival time is only 12–14 months, and the 3-year survival rate ranges between 3–4% [17]. Nearly all GBM patients suffer from a relapse within one year after surgery. Sordillo and colleagues have postulated that radio- and chemotherapy may even induce increased ceramide levels to be metabolized into S1P [18]. They believe that this high S1P to ceramide balance is part of the reason for the nearly 100% recurrence rate. Thus, blocking S1P generation or signaling might be an interesting and effective therapeutic option for GBM. 

In the brain, neuronal and astrocytic cells are able to synthesize and export S1P, and thus represent a source of extracellular S1P [19]. Furthermore, S1P production and secretion has also been described for GBM cells [20]. Several studies argue for a potential role of S1P in the etiology of GBM due to its involvement in diverse cellular processes which are imperative particularly for cancer progression, including invasion, growth, migration, survival, tumor growth, and angiogenesis [21]. These cellular effects of S1P are mediated through the unique family of G protein-coupled receptors (GPCRs) namely S1PR1–S1PR5. The specific effects of S1P in glioblastoma cells are increasingly explored since the early 1990s. Previous reports suggest that S1P is involved in proliferation, migration, and invasion of tumor cells, and glioblastoma stem cell survival [22]. Elevated S1P levels were found in GBM tissues [23] and high levels of SphK1 expression markedly correlated with a shorter survival time of GBM patients [10]. 

In our previous studies, we found that S1P is not only mitogenic in smooth muscle cells [24] and monocytes [25] but it also stimulates the motility and invasiveness of GBM cell lines in vitro. In LN-18 glioblastoma cells, we confirmed the expression of S1P metabolizing enzymes, its receptors, and regulation of cell migration. These findings were accompanied by the poor survival times of GBM patients [10,11]. However, the molecular mechanisms behind the effects of S1P on GBM cells in vitro as well as in vivo remain largely undetermined. In this context, here we summarize updates on current research findings and report the major signaling pathways and intermediates observed in one of the most malignant cancer types, the GBM. Further, the roles of S1P receptors in the spectrum of molecular mechanisms associated with GBM pathobiology are described. 

**Figure 2:**
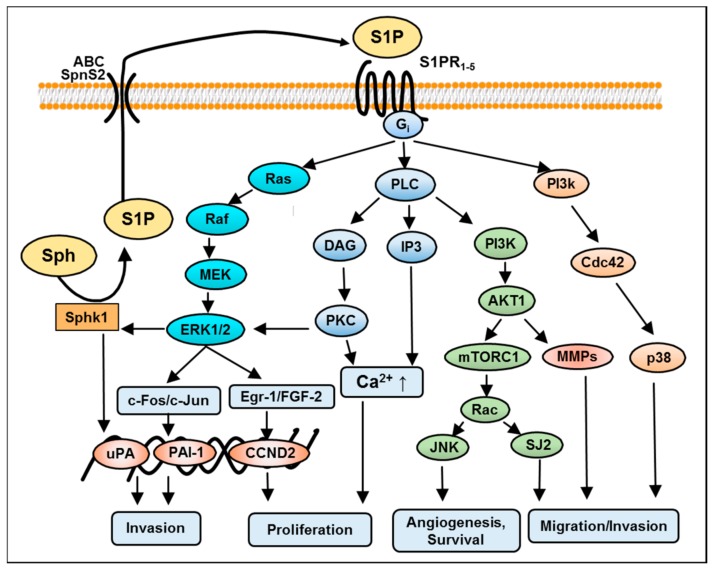
Schematic diagram depicting guanine nucleotide-binding protein (Gi)-mediated S1PRs activation and multiple signaling pathways in glioblastoma. S1PRs via stimulation of the Gi may simultaneously activate MAPK-ERK1/2, c-jun N-terminal kinases (JNK), phospholipase C (PLC), phosphoinositide 3-kinase (PI3K) and p38 pathways in glioma cells. Following the activation of downstream signaling, ERK1/2 facilitates the co-expression and activation of c-jun, c-fos and the early growth response (Egr-1)/fibroblast growth factor (FGF-2) system respectively. Cell cycle arrest by CCND2 (cyclin D2) gene expression and the PLC/Ca^2+^ system is responsible for proliferation. Sphingosine kinase 1 (SphK1) signaling is necessary for the maintenance of urokinase plasminogen activator (uPA) expression and the basal invasive activity of glioma cells by a receptor-independent mechanism. While phosphorylation of SphK1 by ERK1/2 regulates S1P production and spiral signaling PI3k/ATK pathways through the activated downstream targets, mechanistic target of rapamycin (mTOR) and metalloproteases (MMPs) lead to angiogenesis and survival. On the other hand, p38 signaling induces migration. Additional abbreviations used in the figure are defined as follows: DAG, diacylglycerol; ERK1/2, extracellular signal-regulated kinase 1 and 2; Gi, guanine nucleotide-binding protein; IP3, inositol-1, 4, 5-triphosphate; MEK, mitogen-activated protein kinase; PI3K, phosphoinositide 3-kinase; PKC, protein kinase C; PLC, phospholipase C; ↑, increased.

The intracellular downstream effectors of S1P are most fundamental for cancer cell processes, and are not well understood so far in relation to cancer progression. In order to foster effective treatment strategies, the investigation of the signaling pathways and intracellular factors involved in development and progression of GBM cells has been a particular focus in GBM research. Comparable to other cancer types, several studies have investigated downstream mechanisms and biological effects of S1P on glioma cells. Earlier studies have suggested the sphingolipid signaling pathways as potential therapeutic target in gliomas [26]. S1P exerts a variety of responses in GBM cells such as differentiation, proliferation, migration, and survival [27]. These autocrine and paracrine effects are mediated either by extracellular S1PRs or through yet-unknown intracellular targets. S1PR1–S1PR5 bind S1P with high affinity. S1PRs display tissue-specific expression patterns, which subsequently activate multiple Gα protein-coupled pathways, and determine cellular processes responsible for cancer pathology [28]. An overlapping function and an ability of some opposite effects are peculiarities of these receptors. Studies from our laboratory and other investigators suggest that human GBM cells express S1PR1, S1PR2, S1PR3, and S1PR5 [11,29,30]. A recent investigation by Bernhart and colleagues showed that all S1PRs have an impact on proliferation in U87-MG GBM cells [30]. S1P induced a shape change in rat C6 glioma cells acting mainly through the S1PR2 receptor, and co-operatively through the S1PR1/S1PR3 receptors [31]. In GBM tissue specimens, the expression of S1PR1, S1PR2, and S1PR3 was also increased compared to healthy brain tissue, but only S1PR1 and S1PR2 were significantly associated with patients’ survival rates [11,29]. Furthermore, a study by Quint et al. showed S1PR5 to also be a prognostically significant factor for the survival of patients suffering from GBM [32]. However, the role of the individual receptor subtype depends on the activation of the respective downstream effector proteins, in particular coupling to respective G-proteins [33]. For instance, S1PR1, S1PR2 and S1PR5 signal via Gi/o. S1PR2 and S1PR3 activate Gq, and S1PR2, S1PR3, and S1PR5 bind with G12/13. These Gα proteins stimulate the Ras/extracellular signal-regulated kinase (ERK), phosphoinositide 3-kinase (PI3K)/AKT, and Rho/ Ras homolog gene family (RhoA) kinase (ROCK) signaling pathways [28]. In addition, S1PR1 couples via a Gi-protein to multiple effector pathways, including phospholipase C (PLC), adenylate cyclase, and Ras/MAPK (Ras GTPase/mitogen-activated protein kinase) (Figure 2). Based on the in vivo and in vitro studies carried out so far, Table 1 summarizes the role of S1P, its metabolizing enzymes, S1PRs and the signaling cascade involved in GBM.

In GBM, S1P activates multiple signaling pathways in parallel including mitogen-activated extracellular signal-regulated kinase (MAPK/ERK), protein kinase C (PKC), Ca^2+^ signaling via PLC and Phospholipase D (PLD). In the following section, we attempt to elaborate how the individual or simultaneous S1P-mediated signaling cascades participate in cellular responses in GBM progression. 

## 4. S1P-Induced Mitogen-Activated Protein Kinase (MAPK)/Extracellular Signal-Regulated Kinase (ERK) Kinase Signaling in GBM

The activation of the mitogen-activated extracellular signal-regulated kinase cascade occurs via the stimulation of several growth factors, including the epidermal growth factor, the platelet-derived growth factor and the vascular endothelial growth factor [34]. In a comparable manner, S1P receptors are differentially coupled to heterotrimeric G-proteins and therefore the effect of the activation of ERK depends on each subtype of S1PR (e.g., the S1P-induced Gi-coupled receptor requires ERK activation to stimulate glioma cell proliferation and survival [44]). In the U373 GBM cell line, S1P attributes this proliferation through S1PR1, S1PR2, and S1PR3 receptors [45]. The overexpression of these S1PRs subtypes has resulted in ERK activation, consecutive DNA synthesis and associated GBM cell growth [36]. Interestingly, the expression of S1PR5 blocks the proliferation of this glioblastoma cell lines. Another report suggests that S1PR1 and S1PR3 are responsible for promoting migration and cell survival, however, S1PR2 attenuates cells migration [3]. Further results propose that S1PR1 may be the key receptor facilitating stimulation of the ERK/early growth response (Egr-1)/fibroblast growth factor (FGF-2) system in native C6 glioma cells whereas S1PR5 may be responsible for stimulation of PLC-Ca^2+^ system and PLD [46].

Some small adapter proteins also play an additive role in the activation and inactivation of ERK signaling. For example, GluR2 (glutamate receptor 2) overexpression in U87-MG cells inhibits proliferation by inactivating ERK1/2/Src (sarcoma proto-oncogene kinase) phosphorylation, and induces apoptosis and expression of the scaffold protein GRIP (glutamate receptor interacting protein), which is essential for the effect of GluR2 on ERK/Src inactivation [47]. Another enzyme molecule is SHP-2 (Src homology-2 domain-containing phosphatase)—a ubiquitously expressed tyrosine phosphatase—which was shown to preferentially bind to and dephosphorylates Ras to increase its association with Raf and activate downstream proliferative Ras/ERK/MAPK signaling in a mouse model of GBM [48]. Interestingly, oncogenic Ras has been reported to regulate bioactive sphingolipid abundance in a SphK-1-dependent manner, suggesting a mutual interaction between S1P downstream singling and S1P biosynthesis in cancer cells [49]. 

## 5. S1P-Mediated Phosphoinositide 3-Kinase/AKT Pathway in GBM

PI3K/AKT is another signaling pathway involved in GBM cell growth, proliferation, survival, and motility [50]. The serine/threonine kinase AKT, the downstream effector of PI3K, was found to be constitutively active in some glioma cell lines. PI3K/AKT also facilitates motility and survival under stress, which reflects the invasive phenotypic characteristic of GBM [51].

**Figure 3:**
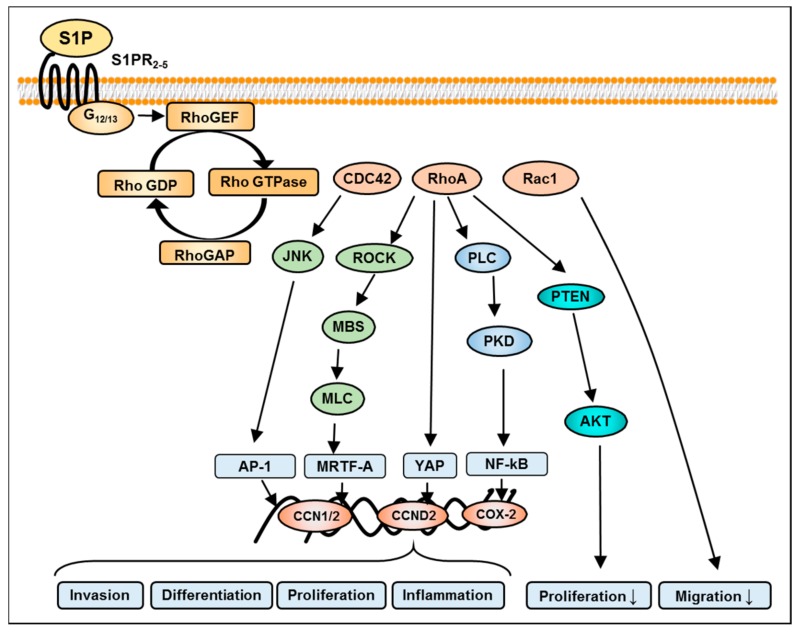
Graphic of S1PR pathways involved in RhoA (Ras homolog gene family, member A) and transcription factor activation in glioma cells. A Rho-like subfamily has been identified which becomes activates via the stimulation of G12/13-protein subunit of S1PRs. The guanine nucleotide exchange factor activates downstream signaling intermediates such as Rho GTPases (guanosine triphosphate hydrolase enzymes) namely CDC42 (cell division control protein 42 homolog), RhoA, and Rac1 (Ras-related C3 botulinum toxin substrate 1). An Rho-kinase-dependent increase in the phosphorylation of the myosin light chain (MLC) by inhibiting the myosin-binding subunit (MBS) causes myofibril reorganization, contraction, and activation of downstream transcriptional effectors. The RhoA-mediated (Ras homolog gene family) transcriptional network involves activator protein 1 (AP1), yes-associated protein (YAP), myocardin-related transcription factor A (MRTF-A), and nuclear factor kappa-light-chain-enhancer of activated B cells (NF-kB). Resulting target genes are involved in the regulation of invasion, proliferation, and differentiation of glioma cells. Inversely, nuclear phosphatase and tensin homolog (PTEN) expression can reduce AKT phosphorylation and consequently inhibit proliferation. On the other hand, Rac1 inhibits migration. Additional abbreviations used in the figure are defined as follows: GEF, guanine nucleotide exchange factor; PKD, protein kinase D; ROCK, RhoA kinase; SRF, serum response factor; ↓inhibition.

In our own studies, to find out which pathway is involved in the S1P-stimulated migration of LN18 GBM cells, specific inhibitors of signaling protein molecules known to be activated by S1P, were utilized. Only the blocking of PI3K/AKT1 signaling by LY294002 completely inhibited S1P stimulated LN18 cell migration. This result was underlined by an amplified phosphorylation of AKT1 after stimulation of the cells with S1P. The PI3K/AKT1 pathway is known to be activated by S1PR1, S1PR2, and S1PR3 via the Gi-coupling of these receptors, of which, AKT is considered as a major downstream signaling molecule of S1PR1. For S1PR1 and S1PR3 a PI3K/AKT-dependent stimulation of cell migration was described, whereby S1PR2 uses this signaling cascade only as a side trail, while G12/13-protein-mediated Rho signaling (as the main pathway; Figure 3) may negatively regulate cell migration [52,53,54]. A PI3K/AKT-dependent increase in invasion of glioma cells was also seen after the stimulation of S1PR2 in a study carried out by Young and Van Brocklyn [36]. This is in agreement with previous results from our own studies, where pharmacological inhibition or specific silencing of S1PR2 resulted in a remarkable reduction of LN18 cell migration [11]. 

Large-scale genomic analyses of GBM have demonstrated that the RTK (receptor tyrosine kinases)/phosphatase and tensin homolog (PTEN)/PI3K/AKT pathway is mutated or overly activated in the majority of GBM subtypes. Thus, activation of AKT and phospho-AKT levels are elevated in the majority of GBM tumor samples and cell lines, enabling these cells to grow in an uncontrolled manner, evade apoptosis, and enhance tumor invasion [55]. Intriguingly, the PI3K/AKT/mechanistic target of rapamycin (mTOR) signaling was elevated in ~88% of all glioblastomas [56]. An association with FAK (focal adhesion kinase) and Src has long been established for PI3K [57], thus linking it to signaling complexes associated with adhesion, motility, and invasion [58]. Further data exhibit a connection between AKT/mTOR activity and GBM motility. This implies a link between two almost ubiquitous features of GBM: high activity of the PI3K signaling cascade and tumor dissemination throughout the whole brain [59].

## 6. S1P-Mediated Activation of Protein Kinase C in GBM

Protein kinase C isozymes are a ubiquitous group of phospholipid-dependent serine/threonine kinases that function in numerous different cell types including cancerous cells. A broad foundation of data has established the central role of PKC isozymes in multiple signal transduction systems as key regulators of cell function, including differentiation, proliferation, survival and motility [60]. PKC isozymes respond to a variety of external stimuli, including growth factors, hormones, and other membrane receptor ligands. In the last three decades, further substantial work has also explored the signaling mechanism(s), regulation and function of PKC isozymes in the progression of multiple cancer types [61]. However, despite the broad actions, and due to the variability of the effects controlled by PKC isoforms, the involvement of this kinase family in the S1P-mediated growth regulation of GBM cells is only inadequately implied [62,63]. 

The role of PKC and its isozymes in the oncogenic regulation has been studied in in-vitro and in-vivo models of high-grade gliomas [64]. PKC isozymes are classified into three categories: conventional, novel and atypical PKCs (Table 2). Each of these subtypes of isoforms appears to play a role in the pathogenesis of glioma formation, however, their cellular effects depend on the upstream signaling cascade. PKCα exerts a mitotic and survival-promoting effect in glioma cell lines. Loss of PKCα was associated with an increased sensitivity to a variety of apoptotic stimuli [65]. PKCβ isoforms have been implicated in the progression of many cancer types, including GBM [66]. In the U251MG cell line, overexpression of PKCγ is associated with increased growth and proliferation through an ERK/Elk-1 (ETS transcription factor-1) pathway [67], whereas PKCδ is associated with decreased growth of GBM cells. The expression of the recently described isoform PKCη correlates with a high degree of proliferation in GBM cell lines [68]. The activation of PKCι promotes motility and invasion, while silencing of PKCι induced a decrease in the proliferation of GBM cells [69]. In in-vitro studies, PKCε expression was found to be elevated by between three to 30 times in GBM cells as compared to the levels in normal human glial cell cultures [70]. Moreover, overexpression of PKCε was detected in histological samples from anaplastic astrocytoma, GBM and gliosarcoma, and is considered an important marker of negative disease outcome [71]. These results suggest that distinctive PKC isoforms have been associated with altered proliferation rates of GBM cells [72]. 

Despite the profound role of various PKC isoforms in GBM, the link between S1P signaling and PKC is only sparely addressed in GBM. It has been demonstrated that PKC regulates SphK1 and increases S1P secretion, thereby allowing its autocrine/paracrine actions [73]. Interestingly, the inhibition of SphK1 in various carcinoma cells (breast, lung and colon) decreased proliferation and cell survival by compromising PKC activity and cytokinesis, which suggests a central PKC-dependent role of S1P in these carcinoma cells [74]. In native C6 glioma cells, stimulation of Gi by S1P activates PKC signaling followed by ERK activation, Egr-1 and FGF-2 expression. This effect of S1P on ERK/Egr-1/FGF-2 is mediated by S1PR1 [31]. S1P, through the ERK/Egr-1/FGF-2 system, also regulates the expression of the urokinase plasminogen activator (uPA), a protein known to stimulate cancer cells’ invasiveness via the S1PR1 receptor in human U118 cells [75]. The role of each of the PKC isozymes, the signaling pathways involved and the findings from several in vivo and in vitro studies are concisely outlined in Table 2.

## 7. S1P-Mediated Activation of Phospholipase C and D in GBM

In recent years, several studies have shown that the activation of phospholipase signaling networks has been involved in cancer growth. It has been shown that S1P increases inositol phosphate levels by activating phospholipase C (PLC) [86]. S1P-induced ERK activation is mediated by at least two signaling pathways (i.e., pertussis toxin (PTX) sensitive and insensitive pathways). In the latter PTX insensitive pathway, the PLC/PKC system plays an essential role.

**Figure 4:**
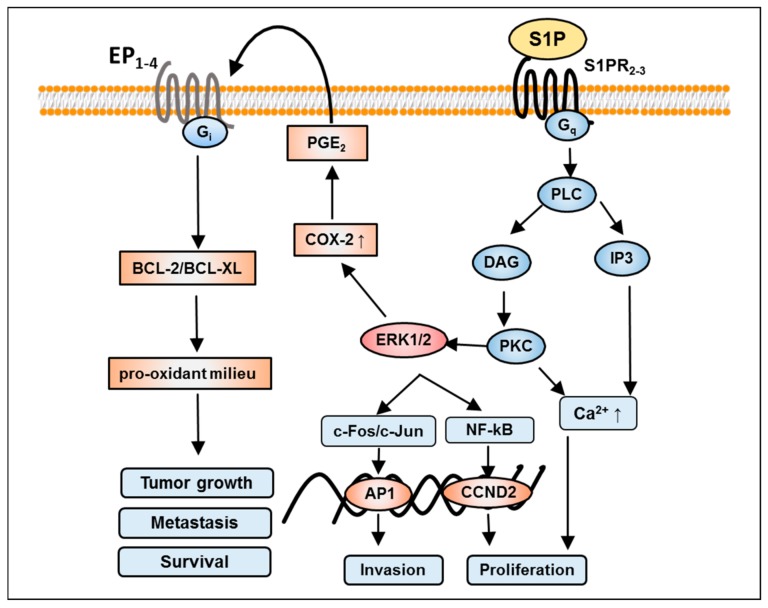
Illustrative scheme of Gq-protein (Gq)-mediated S1P effects in glioblastoma. Activation of S1PR2 and S1PR3 through stimulation of Gq-subunit boosts calcium mobilization and PLC/PKC/ERK1/2 signaling. Following either pathway increases invasion and proliferation by activation of transcription factor activating protein 1 (AP1) and NF-kB-mediated CCND2 expression, respectively. Conversely, S1P also induces cyclo-oxygenase 2 (COX-2) expression and therefore prostaglandin E2 (PGE2) production, in turn, transactivates prostaglandin receptors (EP1-4) receptors leading to a Gi-dependent intracellular signaling mechanism (e.g. activation of the BCL-2/BCL-XL family members). Subsequently, this cascade upturns oxidative stress which is responsible for the transformation of a normal cell to tumor cell, its metastasis and thus survival. Additional abbreviations used in the figure are defined as follows: BCL2; B-cell lymphoma 2; BCL-XL, B-cell lymphoma-extra-large; Gi, Gi-protein; ↑, increased.

In most cases, when the GPCRs are linked to PLC, the PTX insensitive part of the enzyme activation is mediated through the G-proteins [42]. Thus, it is reasonable to assume that S1P may stimulate both signaling pathways mediated by Gi-proteins (Gi) and Gq-proteins (Gq) in GBM cells. Gq-protein-mediated activation of PLC, and transactivation of EP1-4 receptors are schematically depicted in Figure 4. An elevated expression of PLCγ is associated with a worsened prognosis for GBM patients [87]. These results point to a key role for PLCγ activation as a common downstream pathway for growth factor-induced tumor infiltration and as a possible target for anti-invasive therapy in GBM patients. 

S1P has been shown to activate PLC, and subsequently Ca^2+^ mobilization, in several types of cells [88]. Sphingosine activates PLC-dependent pathways and controls Ca^2+^ signals in glioma C6 cells [89]. Furthermore, in glioma C6 cells, S1P also stimulates both the PLC/Ca^2+^ system and PLD which is responsible for proliferation [46]. However, these cellular actions depend on the activation of the respective S1P receptor subtype, for example, S1PR1 appears to be more important than S1PR2 for the stimulation of the ERK/FGF-2 system in rat astrocytes, whereas S1PR2 may be responsible for the activation of the PLC/Ca^2+^ system [90]. Thus, S1PR1 and S1PR2 seem to couple similar types of G-proteins and might share the same signaling pathways but can also favor a specific downstream signaling.

PLD enzymes, namely PLD1 and PLD2, hydrolyze membrane phospholipids, such as phosphatidylcholine (PC), to generate phosphatidic acid (PtdOH), which acts as an important lipid second messenger for GPCR. As a result, both PLD and phosphatidic acid contribute to cancer cell proliferation and survival. Multiple cancer categories, including breast, gastric, and renal cancers, show elevated PLD activity compared with normal tissue [91]. Further, a novel signaling pathway JAK3-Fes-PLD2 (Janus kinase 3-Feline sarcoma oncogene-phospholipase D2) has been found to be responsible for the highly proliferative phenotype of MDA-MB-231 breast cancer cells [92]. Recent advances in the development of isoenzyme-selective PLC/PLD inhibitors suggested that these small molecules may represent promising compounds for the management of certain cancer types [91]. Interestingly, PLD is also activated in different glioma cells (i.e., via phosphorylation by casein kinase-II in human U87 astroglioma cells [93]). Thereby, PLD mediates survival signaling through direct regulation of the AKT pathway in both U87-MG and U118MG GBM cell lines [94]. In addition, overexpression of PLD enhances glioma cell invasion via PKC and PKA (protein kinase A)/nuclear factor kappa-light-chain-enhancer of activated B cells/Sp1-mediated signaling pathways [95].

## 8. S1P-Mediated Activation of Rho Signaling in GBM

GTPases are molecular knobs that regulate a wide variety of signaling pathways in many cell types including glial cells. The principal function of these GTPases is to control the regulation of the actin cytoskeleton. However, their role in the formation of microtubule dynamics, cell polarity, and transcription factor activity is also imperative [96]. Signaling through S1PRs leads to the activation of the G12/13-protein subunit and associated GTPase pathways. RhoA, Rac, Ras and Cdc42 members of the Rho family of GTPases are of particular interest for their roles in cell migration [97] (Figure 4). In GMB, it has been shown that RhoA and Rac are both required for establishing cell polarity, and excessive Rho activity appears to inhibit motility and polarization [98]. In particular, Rho mediates stress fiber formation through a phosphorylation of Rho kinase, which hinders the activity of myosin light-chain phosphatase thereby stabilizing actin filaments [53]. 

The Rho signaling pathway is involved in the inhibition of GBM cell migration through S1PR2, which is linked with the PTEN-independent activation of RhoA and suppression of Rac1 [54]. On the other hand, Sanchez et al. have reported that S1PR2-mediated inhibition of glioma cell migration occurs via PTEN activation, which interrupts adherens junctions [99]. However, the PTEN gene has been found to be mutated in 15% to 40% of GBM cases [16], which supports additional signaling pathways being relevant in controlling GBM cell migration in these tumors. Another study also suggested that S1P-mediated activation of G12/13-Rho by S1PR2 leads to the inhibition of Rac [100]. Studies carried out by Takuwa et al. have supported the concept that inhibition metastasis and tumor cell migration occurs as a result of S1P2-mediated down-regulation of cellular Rac but, unexpectedly, enhances glioma cell invasiveness by stimulating cell adhesion [36]. In contrast, Lepley et al. demonstrated that S1P2-specific inhibition of Rac activity is not involved in the S1P-mediated inhibition of migration in the human glioblastoma cell lines [53]. This receptor also impedes growth factors induced cell migration such as PDGF, IGF, and chemokines [101]. Further, Rho/ROCK signaling, which is involved in GBM cell proliferation and migration, may be interrelated with ERK signaling [102]. In addition to the Rho GTPases themselves, several of their downstream and upstream regulators comprising guanine nucleotide exchange factors (GEFs), GTPase-activating proteins (GAPs), PI3K, and PTEN have also been implicated in primary brain tumors including GBM [103]. S1PR2 has an ability to enhance the expression of plasminogen activator inhibitor-1 (PAI-1) and urokinase-type plasminogen activator receptor (uPAR) through the activation of dual downstream signaling cascades MEK1/2 and Rho-kinase, which are vital for glioblastoma invasiveness. At the transcriptional level, S1PR2-Rho responses are not well understood, but the study carried out by Yu and group shows that Rho GTPase-coupled S1P receptors can trigger the Hippo signaling pathway and its allied transcriptional effectors [104].

## 9. Summary

Altogether, evidence from diverse in vitro and in vivo studies indicate that S1P, its metabolizing enzymes, and S1PRs play significant roles in GBM cell fate determination. Numerous studies demonstrated that activation of S1PRs-mediated intracellular signaling pathways establish functional actions of S1P utilized by GBM. Despite the fact that the signaling mechanisms of each S1PR subtype have been fairly well recognized, their functional roles in GBM are still inadequately understood. Further, although convincing implications are available for the role of S1P in GBM proliferation and survival, invasion, migration, and metastasis, there are still some discrepancies about the impact of S1P signaling on the molecular mechanisms of GBMs due to their heterogeneity. An alternative reason is the complex glioblastoma microenvironment which is dynamically regulated by intra- and inter-cellular signaling cascades. The involvement of small proteins like mTORC1, metalloproteases, FGF-2 and NF-kB promotes tumor growth and invasion by enduring glioblastoma stem cells, thereby promoting angiogenesis. Therefore, there is much to be discovered about the benefits and risks of inhibiting the different small molecules and effector proteins involved in multiple tumorigenic pathways. Further clinical studies are essential to finding a promising alternative to treat GBM patients rather than the conventional treatment. Last, but not least, a broader understanding of the mechanisms of S1P signaling in GBM progression and a more sophisticated experimental system are required in order to develop effective treatment strategies.

## Figures and Tables

**Table 1:** Summary of S1P enzyme(s) or receptor(s)-mediated effects in GBM.

Models (in vitro/in vivo)	Involved Enzyme(s) or S1P Receptor(s)	Signaling Pathway(s)	Findings
LN-18 U87-MG	↑ SphK1, ↑ S1PR1-3	PI3K/AKT1 activation	poor prognosis and survival of GBM patients; inhibition of SphK1 reduces cell viability; inhibition of S1PR1/2 diminishes cell migration [11]
U373-MG	↑ S1PR1-3 expression	MAPK/ERK and PI3Kβ pathway	S1P stimulates glioma cell proliferation [34]
U373-MG, GBM-6, and GBM-12	↑ SphK1 S1PR2	MEK1/2 and Rho/ROCK	S1P induced ↑ mRNA and protein expression of PAI-1 and uPAR is correlated with invasion of glioblastoma cells [35]
U373-MG U118-MG	↑ S1PR1, S1PR2, and S1PR3	MAPK-ERK Rho/ROCK ↑ CCN1/Cyr61protein expression	S1P stimulates growth and invasiveness; S1PR2-mediates migration, and invasiveness; ↑ CCN1/Cyr61 linked to tumor cell adhesion and angiogenesis [36]
U87-MG	S1P	MAPK-ERK Rho/ROCK; PLC; MT1-MMP/G6PT	S1P induces Ca^2+^ mobilization via MT1-MMP/ G6PT axis which is responsible for infiltrative and invasive properties of GBM [37]
LN-229 and U373-MG xenografts	SphK1	AKT/JNK c-jun/ATF2 transcription	↓ SphK1 expression or its inhibition by SK1-I reduces growth, migration and invasion of glioma cells *in vitro* and *in vivo* [38]
Rat C6 glioma	S1PR2	MAPK/ERK, PKC and PLC/D Ca^2+^ signaling	S1P receptors are linked to at least two signaling pathways, i.e., the PTX-sensitive Gi /Go-protein pathway and the toxin-insensitive Gq/G11-phospholipase C-PKC pathway [39]
Rat C6 glioma, 1321-N1 astrocytoma cells	S1P, S1PR2	PI3K/Cdc42/p38MAPK and PI3K/Rac1/JNK	S1P, through S1PR2, negatively regulated the migration [40]
1321N1	S1P levels	G_12/13_-mediated Rho/ROCK activation	S1P enhanced proliferation by activation of both MRTF-A and YAP [41]
U-373 MG, U87-MG, M059K, U-1242 and A172	S1P levels ↑ S1PR1-3	MAPK/ERK and PI3K	S1P enhances glioma cell motility and invasiveness via Gi-coupling [42]
T98G and G112 cells	S1P1, S1P2, S1P3 and S1P5	PTEN/AKT/Egr	↓ S1PR1 expression enhances the malignancy of glioblastoma; ↑ proliferation correlates with the shorter survival of patients with GBM [43]
human glioblastoma specimens	S1PR1	NS	low expression of S1PR1 was significantly correlated with the high MIB-1 LI in glioblastomas from patients showing reduced survival [29]
U87-MG	S1P levels	NS	SphK1 inhibition reduced angiogenesis in a co-culture *in vitro* model [23]
glioblastoma primary tumors glioma specimens	↑ S1P and SphK1 levels and ↓ SPP2 expression	NS	↑ S1P and *↓* ceramide content; high SphK1 and low SPP2 observed [23]

Abbreviations used in the table are defined as follows: AKT, AKT8 virus oncogene cellular homolog (also known as protein kinase B); G6PT, glucose 6-phosphatase; MT1-MMP, membrane-type-1 matrix metalloproteinase; NS, not studied; PAI-1, plasminogen activator inhibitor-1; SK1-I, sphingosine kinase 1-inhibitor; SPP2, S1P phosphatase 2; uPAR, urokinase-type plasminogen activator receptor; ↑, increased; ↓, reduced.

**Table 2:** Summary of PKC isozymes and their roles in multiple types of glioblastoma cells.

Class	Type of Isoform	Model (*in vitro/in vivo*)	Signaling Pathway(s)	Findings
conventional isoforms	α	Rat C6 glioma, U87MG	ERK1/2 activation	cell proliferation, survival, invasion, migration [65,76]
	βI	U87-MG, T98G xenografts	AKT/GSK3β and S6 kinase	regulation of cell growth [66,77]
	βII	U87-MG	CAK activation	cell cycle activation [78]
	γ	human glioblastoma cells	NS	expression of PKCγ in GBM cells [72]
novel isoforms	δ	U373-MG, A172 U87-MG, T98G	EFG/Src and SphK1, elevated PAI-1 levels; c-MET/NOTCH2	Cell motility, invasion and infiltration [79,80]
	ε	GBM specimens and gliosarcoma samples	PI3K/AKT pathway	Overexpression in primary GBM tumors; regulates the apoptosis and survival [71]
	θ	U-251 and 5310 xenograft cell lines	MAPK/ERK signaling	Proapoptotic kinase; radioresistance [81]
	η	U-1242 and U-251	AKT/mTOR and Erk/Elk-1 signaling	cell proliferation [68,82]
	μ	human glioblastoma cell lines	NS	proapoptotic kinase [83]
atypical isoform	ι/λ	U87MG and gliomas	PI3K signaling	cell proliferation, motility and invasion [69,84]
	ζ	U251 cell line and the 5310 xenograft glioma cell line	RhoA-dependent PKCζ/Raf1/MEK/ERK	cell proliferation, survival, invasion, and migration [81,85]

Abbreviations used in the table are defined as follows: GSK3β, Glycogen synthase kinase 3β; S6K, ribosomal S6 protein kinase; c-MET, tyrosine-protein kinase Met (mesenchymal epithelial transition); NS, not studied; NOTCH2, Neurogenic locus notch homolog protein 2; mTOR, mechanistic target of rapamycin; EFG, elongation factor G; CAK, CDK (cyclin-dependent kinase)-activating kinase.

## References

[1] Pyne N.J., Pyne S. (2010). Sphingosine 1-phosphate and cancer. Nat. Rev. Cancer.

[2] Ponnusamy S., Meyers-Needham M., Senkal C.E., Saddoughi S.A., Sentelle D., Selvam S.P., Salas A., Ogretmen B. (2010). Sphingolipids and cancer: Ceramide and sphingosine-1-phosphate in the regulation of cell death and drug resistance. Future Oncol..

[3] Pyne N.J., Ohotski J., Bittman R., Pyne S. (2014). The role of sphingosine 1-phosphate in inflammation and cancer. Adv. Biol. Regul..

[4] Oskouian B., Saba J. (2007). Sphingosine-1-phosphate metabolism and intestinal tumorigenesis: Lipid signaling strikes again. Cell Cycle.

[5] Tilly J.L., Kolesnick R.N. (2002). Sphingolipids, apoptosis, cancer treatments and the ovary: Investigating a crime against female fertility. BBA-Mol. Cell. Biol. Lipids.

[6] Sutphen R., Xu Y., Wilbanks G.D., Fiorica J., Grendys E.C., LaPolla J.P., Arango H., Hoffman M.S., Martino M., Wakeley K. (2014). Lysophospholipids are potential biomarkers of ovarian cancer. Cancer Epidemiol Biomark. Prev..

[7] Watson C., Long J.S., Orange C., Tannahill C.L., Mallon E., McGlynn L.M., Pyne S., Pyne N.J., Edwards J. (2010). High expression of sphingosine 1-phosphate receptors, S1P1 and S1P3, sphingosine kinase 1, and extracellular signal-regulated kinase-1/2 is associated with development of tamoxifen resistance in estrogen receptor-positive breast cancer patients. Am. J. Pathol..

[8] Takabe K., Spiegel S. (2014). Export of sphingosine-1-phosphate and cancer progression. J. Lipid Res..

[9] Sarkar S., Maceyka M., Hait N.C., Paugh S.W., Sankala H., Milstien S., Spiegel S. (2005). Sphingosine kinase 1 is required for migration, proliferation and survival of MCF-7 human breast cancer cells. FEBS Lett..

[10] Van Brocklyn J.R., Jackson C.A., Pearl D.K, Kotur M.S., Snyder P.J., Prior T.W. (2005). Sphingosine kinase-1 expression correlates with poor survival of patients with glioblastoma multiforme: Roles of sphingosine kinase isoforms in growth of glioblastoma cell lines. J. Neuropathol. Exp. Neurol..

[11] Bien-Möller S., Lange S., Holm T., Böhm A., Paland H., Küpper J., Herzog S., Weitmann K., Havemann C., Vogelgesang S. (2016). Expression of S1P metabolizing enzymes and receptors correlate with survival time and regulate cell migration in glioblastoma multiforme. Oncotarget.

[12] Obinata H., Hla T. (2012). Sphingosine 1-phosphate in coagulation and inflammation. Semin. Immunopathol..

[13] Heffernan-Stroud L.A., Obeid L.M. (2013). Sphingosine kinase 1 in cancer. Adv. Cancer Res..

[14] Le Stunff H., Milstien S., Spiegel S. (2004). Generation and metabolism of bioactive sphingosine-1-phosphate. J. Cell. Biochem..

[15] Mora R., Dokic I., Kees T., Hüber C.M., Keitel D., Geibig R., Brügge B., Zentgraf H., Brady N.R, Régnier-Vigouroux A. (2010). Sphingolipid rheostat alterations related to transformation can be exploited for specific induction of lysosomal cell death in murine and human glioma. Glia.

[16] Ohgaki H., Kleihues P. (2007). Genetic pathways to primary and secondary glioblastoma. Am. J. Pathol..

[17] Rulseh A.M., Keller J., Klener J., Šroubek J., Dbalý V., Syrůček M., Tovaryš F., Vymazal J. (2012). Long-term survival of patients suffering from glioblastoma multiforme treated with tumor-treating fields. World J. Surg. Oncol..

[18] Sordillo L.A., Sordillo P.P., Helson L. (2016). Sphingosine kinase inhibitors as maintenance therapy of glioblastoma after ceramide-induced response. Anticancer Res..

[19] Bassi R., Anelli V., Giussani P., Tettamanti G., Viani P., Riboni L. (2006). Sphingosine-1-phosphate is released by cerebellar astrocytes in response to bFGF and induces astrocyte proliferation through G1-protein-coupled receptors. Glia.

[20] Anelli V., Gault C.R., Cheng A.B., Obeid L.M. (2008). Sphingosine kinase 1 is up-regulated during hypoxia in U87-MG glioma cells role of hypoxia-inducible factors 1 and 2. J. Biol. Chem..

[21] Strub G.M., Maceyka M., Hait N.C., Milstien S., Spiegel S. (2010). Extracellular and Intracellular Actions of Sphingosine-1-Phosphate. Adv. Exp. Med. Biol..

[22] Marfia G., Campanella R., Navone S.E., Di Vito C., Riccitelli E., Hadi L.A., Bornati A., de Rezende G., Giussani P., Tringali C. (2014). Autocrine/paracrine sphingosine-1-phosphate fuels proliferative and stemness qualities of glioblastoma stem cells. Glia.

[23] Abuhusain H.J., Matin A., Qiao Q., Shen H., Kain N., Day B.W., Stringer B.W., Daniels B., Laaksonen M.A., Teo C. (2013). Metabolic Shift Favoring Sphingosine 1-Phosphate at the Expense of Ceramide Controls Glioblastoma Angiogenesis. J. Biol. Chem..

[24] Böhm A., Flößer A., Ermler S., Fender A.C., Lüth A., Kleuser B., Schrör K., Rauch B.H. (2013). Factor-Xa-induced mitogenesis and migration require sphingosine kinase activity and S1P formation in human vascular smooth muscle cells. Cardiovasc. Res..

[25] Mahajan-Thakur S., Sostmann B.D., Fender A.C., Behrendt D., Felix S.B., Schrör K., Rauch B.H. (2014). Sphingosine-1-phosphate induces thrombin receptor PAR-4 expression to enhance cell migration and COX-2 formation in human monocytes. J. Leukocyte Biol..

[26] Van Brocklyn J.R. (2007). Sphingolipid signaling pathways as potential therapeutic targets in gliomas. Mini Rev. Med. Chem..

[27] Hla T. (2003). Signaling and biological actions of sphingosine 1-phosphate. Pharmacol. Res..

[28] Rosen H., Goetzl E.J. (2005). Sphingosine 1-phosphate and its receptors: An autocrine and paracrine network. Nat. Rev. Immunol..

[29] Yoshida Y., Nakada M., Sugimoto N., Harada T., Hayashi Y., Kita D., Uchiyama N., Hayashi Y., Yachie A., Takuwa Y. (2010). Sphingosine-1-phosphate receptor type 1 regulates glioma cell proliferation and correlates with patient survival. Int. J. Cancer.

[30] Bernhart E., Damm S., Wintersperger A., Nusshold C., Brunner A.M., Plastira I., Rechberger G., Reicher H., Wadsack C., Zimmer A. (2015). Interference with distinct steps of sphingolipid synthesis and signaling attenuates proliferation of U87-MG glioma cells. Biochem. Pharmacol..

[31] Kim K., Kim Y.L., Sacket S.J., Kim H.L., Han M., Park D.S., Lee B.K., Lee W.K., Ha H.J., Im D.S. (2007). Sphingosine 1-phosphate (S1P) induces shape change in rat C6 glioma cells through the S1P2 receptor: Development of an agonist for S1P receptors. J. Pharm. Pharmacol..

[32] Quint K., Stiel N., Neureiter D., Schlicker H.U., Nimsky C., Ocker M., Strik H., Kolodziej M.A. (2014). The role of sphingosine kinase isoforms and receptors S1P1, S1P2, S1P3, and S1P5 in primary, secondary, and recurrent glioblastomas. Tumour Biol..

[33] Siehler S., Manning D.R. (2002). Pathways of transduction engaged by sphingosine 1-phosphate through G protein-coupled receptors. BBA-Mol. Cell. Biol. Lipids.

[34] Rekers H., Sminia P., Peters G.J. (2011). Towards tailored therapy of glioblastoma multiforme. J. Chemother..

[35] Bryan L., Paugh B.S., Kapitonov D., Wilczynska K.M., Alvarez S.M., Singh S.K., Milstien S., Spiegel S., Kordula T. (2008). Sphingosine-1-phosphate and interleukin-1 independently regulate plasminogen activator inhibitor-1 and urokinase-type plasminogen activator receptor expression in glioblastoma cells: Implications for invasiveness. Mol. Cancer Res..

[36] Young N., Van Brocklyn J.R. (2007). Roles of sphingosine-1-phosphate (S1P) receptors in malignant behavior of glioma cells. Differential effects of S1P2 on cell migration and invasiveness. Exp. Cell Res..

[37] Fortier S., Labelle D., Sina A., Moreau R., Annabi B. (2008). Silencing of the MT1-MMP/G6PT axis suppresses calcium mobilization by sphingosine-1-phosphate in glioblastoma cells. FEBS Lett..

[38] Kapitonov D., Allegood J.C., Mitchell C., Hait N.C., Almenara J.A., Adams J.K., Zipkin R.E., Dent P., Kordula T., Milstien S., Spiegel S. (2009). Targeting sphingosine kinase 1 inhibits Akt signaling, induces apoptosis, and suppresses growth of human glioblastoma cells and xenografts. Cancer Res..

[39] Sato K., Tomura H., Igarashi Y., Ui M., Okajima F. (1999). Possible involvement of cell surface receptors in sphingosine 1-phosphate-induced activation of extracellular signal-regulated kinase in C6 glioma cells. Mol. Pharmacol..

[40] Malchinkhuu E., Sato K., Horiuchi Y., Mogi C., Ohwada S., Ishiuchi S., Saito N., Kurose H., Tomura H., Okajima F. (2005). Role of p38 mitogen-activated kinase and c-Jun terminal kinase in migration response to lysophosphatidic acid and sphingosine-1-phosphate in glioma cells. Oncogene.

[41] Yu O.M., Miyamoto S., Brown J.H. (2015). Myocardin-Related transcription factor A and yes-associated protein exert dual control in G protein-coupled receptor- and RhoA-mediated transcriptional regulation and cell proliferation. Mol. Cell Biol..

[42] Van Brocklyn J.R., Young N., Roof R. (2003). Sphingosine-1-phosphate stimulates motility and invasiveness of human glioblastoma multiforme cells. Cancer Lett..

[43] Yoshida Y., Nakada M., Harada T., Tanaka S., Furuta T., Hayashi Y., Kita D., Uchiyama N., Hayashi Y., Hamada J. (2010). The expression level of sphingosine-1-phosphate receptor type 1 is related to MIB-1 labeling index and predicts survival of glioblastoma patients. J. Neurooncol..

[44] Van Brocklyn J., Letterle C., Snyder P., Prior T. (2002). Sphingosine-1-phosphate stimulates human glioma cell proliferation through Gi-coupled receptors: Role of ERK MAP kinase and phosphatidylinositol 3-kinase beta. Cancer Lett..

[45] Hu W.M., Li L., Jing B.Q., Zhao Y.S., Wang C.L., Feng L., Feng L., Xie Y.E. (2010). Effect of S1P5 on proliferation and migration of human esophageal cancer cells. World J. Gastroenterol..

[46] Sato K., Ui M., Okajima F. (2000). Differential roles of Edg-1 and Edg-5, sphingosine 1-phosphate receptors, in the signaling pathways in C6 glioma cells. Brain Res. Mol. Brain Res..

[47] Beretta F., Bassani S., Binda E., Verpelli C., Bello L., Galli R., Passafaro M. (2009). The GluR2 subunit inhibits proliferation by inactivating Src-MAPK signalling and induces apoptosis by means of caspase 3/6-dependent activation in glioma cells. Eur. J. Neurosci..

[48] Bunda S., Burrell K., Heir P., Zeng L., Alamsahebpour A., Kano Y., Raught B., Zhang Z.Y., Zadeh G., Ohh M. (2015). Inhibition of SHP2-mediated dephosphorylation of Ras suppresses oncogenesis. Nat. Commun..

[49] Gault C.R., Eblen S.T., Neumann C.A., Hannun Y.A., Obeid L.M. (2012). Oncogenic K-Ras regulates bioactive sphingolipids in a sphingosine kinase 1-dependent manner. J. Biol. Chem..

[50] Yuan T.L., Cantley L.C. (2008). PI3K pathway alterations in cancer: Variations on a theme. Oncogene.

[51] Westhoff M.A., Karpel-Massler G., Brühl O., Enzenmüller S., La Ferla-Brühl K., Siegelin M.D., Nonnenmacher L., Debatin K.M. (2014). A critical evaluation of PI3K inhibition in Glioblastoma and Neuroblastoma therapy. Mol. Cell. Ther..

[52] Takuwa N., Du W., Kaneko E., Okamoto Y., Yoshioka K., Takuwa Y. (2011). Tumor-suppressive sphingosine-1-phosphate receptor-2 counteracting tumor-promoting sphingosine-1-phosphate receptor-1 and sphingosine kinase 1-Jekyll Hidden behind Hyde. Am. J. Cancer Res..

[53] Lepley D., Paik J.H., Hla T., Ferrer F. (2005). The G protein-coupled receptor S1P2 regulates Rho/Rho kinase pathway to inhibit tumor cell migration. Cancer Res..

[54] Malchinkhuu E., Sato K., Maehama T., Mogi C., Tomura H., Ishiuchi S., Yoshimoto Y., Kurose H., Okajima F. (2008). S1P(2) receptors mediate inhibition of glioma cell migration through Rho signaling pathways independent of PTEN. Biochem. Biophys. Res. Commun..

[55] McDowell K.A., Riggins G.J., Gallia G.L. (2011). Targeting the AKT pathway in glioblastoma. Curr. Pharm. Des..

[56] Chin L., Meyerson M., Aldape K., Bigner D., Mikkelsen T., VandenBerg S., Kahn A., Penny R., Ferguson M.L., Gerhard D.S. (2008). Comprehensive genomic characterization defines human glioblastoma genes and core pathways. Nature.

[57] Chen H.C., Appeddu P.A., Isoda H., Guan J.L. (1996). Phosphorylation of tyrosine 397 in focal adhesion kinase is required for binding phosphatidylinositol 3-kinase. J. Biol. Chem..

[58] Westhoff M.A., Bruhl O., Nonnenmacher L., Karpel-Massler G., Debatin K.M. (2014). Killing me softly-future challenges in apoptosis research. Int. J. Mol. Sci..

[59] Gulati N., Karsy M., Albert L., Murali R., Jhanwar-Uniyal M. (2009). Involvement of mTORC1 and mTORC2 in regulation of glioblastoma multiforme growth and motility. Int. J. Oncol..

[60] Do Carmo A., Patricio I., Cruz M.T., Carvalheiro H., Oliveira C.R., Lopes M.C. (2010). CXCL12/CXCR4 promotes motility and proliferation of glioma cells. Cancer Biol. Ther..

[61] Garg R., Benedetti L.G., Abera M.B., Wang H., Abba M., Kazanietz M.G. (2014). Protein kinase C and cancer: What we know and what we do not. Oncogene.

[62] Bazzi M.D., Nelsestuen G.L. (1987). Mechanism of protein kinase C inhibition by sphingosine. Biochem. Biophys. Res. Commun..

[63] Hannun Y.A., Bell R.M. (1989). Regulation of protein kinase C by sphingosine and lysosphingolipids. Clin. Chim. Acta.

[64] Bredel M., Pollack I.F. (1997). The role of protein kinase C (PKC) in the evolution and proliferation of malignant gliomas, and the application of PKC inhibition as a novel approach to anti-glioma therapy. Acta Neurochir..

[65] Cameron A.J., Procyk K.J., Leitges M., Parker P.J. (2008). PKC alpha protein but not kinase activity is critical for glioma cell proliferation and survival. Int. J. Cancer.

[66] Teicher B.A., Menon K., Alvarez E., Galbreath E., Shih C., Faul M. (2001). Antiangiogenic and antitumor effects of a protein kinase Cbeta inhibitor in human T98G glioblastoma multiforme xenografts. Clin. Cancer Res..

[67] Mishima K., Ohno S., Shitara N., Yamaoka K., Suzuki K. (1994). Opposite effects of the overexpression of protein kinase Cγ and δ on the growth properties of human glioma cell line U251 MG. Biochem. Biophys. Res. Commun..

[68] Uht R.M., Amos S., Martin P.M., Riggan A.E., Hussaini I.M. (2007). The protein kinase C-*η* isoform induces proliferation in glioblastoma cell lines through an ERK/Elk-1 pathway. Oncogene.

[69] Patel R., Win H., Desai S., Patel K., Matthews J.A., Acevedo-Duncan M. (2008). Involvement of PKC-iota in glioma proliferation. Cell Prolif..

[70] Xiao H., Goldthwait D.A., Mapstone T. (1994). A search for glial expression in tumors of the central nervous system. Pediatr. Neurosurg..

[71] Sharif T.R., Sharif M. (1999). Overexpression of protein kinase C epsilon in astroglial brain tumor derived cell lines and primary tumor samples. Int. J. Oncol..

[72] Xiao H., Goldthwait D.A., Mapstone T. (1994). The identification of four protein kinase C isoforms in human glioblastoma cell lines: PKC alpha, gamma, epsilon, and zeta. J. Neurosurg..

[73] Johnson K.R., Becker K.P., Facchinetti M.M., Hannun Y.A., Obeid L.M. (2002). PKC-dependent activation of sphingosine kinase 1 and translocation to the plasma membrane extracellular release of sphingosine-1-phosphate induced by phorbol 12-myristate 13-acetate(PMA). J. Biol. Chem..

[74] Kotelevets N., Fabbro D., Huwiler A., Zangemeister-Wittke U. (2012). Targeting sphingosine kinase 1 in carcinoma cells decreases proliferation and survival by compromising PKC activity and cytokinesis. PLoS ONE.

[75] Young N., Pearl D.K., Van Brocklyn J.R. (2009). Sphingosine-1-phosphate regulates glioblastoma cell invasiveness through the urokinase plasminogen activator system and CCN1/Cyr61. Mol. Cancer Res..

[76] Hu J.G., Wang X.F., Zhou J.S., Wang F.C., Li X.W., Lü H.Z. (2010). Activation of PKCα is required for migration of C6 glioma cells. Acta Neurobiol. Exp..

[77] Graff J.R., McNulty A.M., Hanna K.R., Konicek B.W., Lynch R.L., Bailey S.N., Banks C., Capen A., Goode R., Lewis J.E. (2005). The protein kinase Cβ-selective inhibitor, Enzastaurin (LY317615.HCl), suppresses signaling through the AKT pathway, induces apoptosis, and suppresses growth of human colon cancer and glioblastoma xenografts. Cancer Res..

[78] Acevedo-Duncan M., Patel R., Whelan S., Bicaku E. (2002). Human glioma PKC-iota and PKC-βII phosphorylate cyclin-dependent kinase activating kinase during the cell cycle. Cell Proliferat..

[79] Paugh B.S., Paugh S.W., Bryan L., Kapitonov D., Wilczynska K.M., Gopalan S.M., Rokita H., Milstien S., Spiegel S., Kordula T. (2008). EGF regulates plasminogen activator inhibitor-1 (PAI-1) by a pathway involving c-Src, PKCδ, and sphingosine kinase 1 in glioblastoma cells. FASEB J..

[80] Hwang E., Yoo K.C., Kang S.G., Kim R.K., Cui Y.H., Lee H.J., Kim M.J., Lee J.S., Kim I.G., Suh Y. (2016). PKCδ activated by c-MET enhances infiltration of human glioblastoma cells through NOTCH2 signaling. Oncotarget.

[81] Alapati K., Gopinath S., Malla R.R., Dasari V.R., Rao J.S. (2012). uPAR and cathepsin B knockdown inhibits radiation-induced PKC integrated integrin signaling to the cytoskeleton of glioma-initiating cells. Int. J. Oncol..

[82] Aeder S.E., Martin P.M., Soh J.W., Hussaini I.M. (2004). PKC-η mediates glioblastoma cell proliferation through the Akt and mTOR signaling pathways. Oncogene.

[83] Okhrimenko H., Lu W., Xiang C., Hamburger N., Kazimirsky G., Brodie C. (2005). Protein kinase C-epsilon regulates the apoptosis and survival of glioma cells. Cancer Res..

[84] Baldwin R.M., Parolin D.A., Lorimer I.A. (2008). Regulation of glioblastoma cell invasion by PKCι and RhoB. Oncogene.

[85] Guo H., Gu F., Li W., Zhang B., Niu R., Fu L., Zhang N., Ma Y. (2009). Reduction of protein kinase C ζ inhibits migration and invasion of human glioblastoma cells. J. Neurochem..

[86] Mattie M., Brooker G., Spiegel S. (1994). Sphingosine-1-phosphate, a putative second messenger, mobilizes calcium from internal storesvia an inositol trisphosphate-independent pathway. J. Biol. Chem..

[87] Mawrin C., Diete S., Treuheit T., Kropf S., Vorwerk C.K., Boltze C., Kirches E., Firsching R., Dietzmann K. (2003). Prognostic relevance of MAPK expression in glioblastoma multiforme. Int. J. Oncol..

[88] Okajima F., Tomura H., Sho K., Nochi H., Tamoto K., Kondo Y. (1996). Involvement of pertussis toxin-sensitive GTP-binding proteins in sphingosine 1-phosphate-induced activation of phospholipase C-Ca^2+^ system in HL60 leukemia cells. FEBS Lett..

[89] Czajkowski R., Sabala P., Baranska J. (1997). Sphingosine modulates Ca signals via phospholipase C dependent pathway in glioma C6 cells. Acta Neurobiol. Exp..

[90] Malchinkhuu E., Sato K., Muraki T., Ishikawa K., Kuwabara A., Okajima F. (2003). Assessment of the role of sphingosine 1-phosphate and its receptors in high-density lipoprotein-induced stimulation of astroglial cell function. Biochem. J..

[91] Brown H.A., Thomas P.G., Lindsley C.W. (2017). Targeting phospholipase D in cancer, infection and neurodegenerative disorders. Nat. Rev. Drug Discov..

[92] Ye Q., Kantonen S., Henkels K.M., Gomez-Cambronero J. (2013). A new signaling pathway (JAK-Fes-phospholipase D) that is enhanced in highly proliferative breast cancer cells. J. Biol. Chem..

[93] Ahn B.H., Min G., Bae Y.S., Bae Y.S., Min D.S. (2006). Phospholipase D is activated and phosphorylated by casein kinase-II in human U87 astroglioma cells. Exp. Mol. Med..

[94] Bruntz R.C., Taylor H.E., Lindsley C.W., Brown H.A (2014). Phospholipase D2 mediates survival signaling through direct regulation of Akt in glioblastoma cells. Pharmacol. Rev..

[95] Park M.H., Ahn B.H., Hong Y.K., Min S. (2009). Overexpression of phospholipase D enhances matrix metalloproteinase-2 expression and glioma cell invasion via protein kinase C and protein kinase A/NF-kappaB/Sp1-mediated signaling pathways. Carcinogenesis.

[96] Etienne-Manneville S., Hall A. (2002). Rho GTPases in cell biology. Nature.

[97] Radeff-Huang J., Seasholtz T.M., Matteo R.G., Brown J.H. (2004). G protein mediated signaling pathways in lysophospholipid induced cell proliferation and survival. J. Cell. Biochem..

[98] Xu J., Wang F., Van Keymeulen A., Herzmark P., Straight A., Kelly K., Takuwa Y., Sugimoto N., Mitchison T., Bourne H.R. (2003). Divergent signals and cytoskeletal assemblies regulate self-organizing polarity in neutrophils. Cell.

[99] Sanchez T., Thangada S., Wu M.T, Kontos C.D, Wu D., Wu H., Hla T. (2005). PTEN as an effector in the signaling of antimigratory G protein-coupled receptor. Proc. Natl. Acad. Sci. USA.

[100] Sugimoto N., Takuwa N., Okamoto H., Sakurada S., Takuwa Y. (2003). Inhibitory and stimulatory regulation of Rac and cell motility by the G12/13-Rho and Gi pathways integrated downstream of a single G protein-coupled sphingosine-1-phosphate receptor isoform. Mol. Cell Biol..

[101] Michaud J., Im D.S., Hla T. (2010). Inhibitory role of sphingosine 1-phosphate receptor 2 in macrophage recruitment during inflammation. J. Immunol..

[102] Zohrabian V.M., Forzani B., Chau Z., Murali R., Jhanwar-Uniyal M. (2009). Rho/ROCK and MAPK signaling pathways are involved in glioblastoma cell migration and proliferation. Anticancer Res..

[103] Khalil B.D., El-Sibai M. (2012). Rho GTPases in primary brain tumor malignancy and invasion. J Neurooncol..

[104] Yu F.X., Zhao B., Panupinthu N., Jewell J.L., Lian I., Wang L.H., Zhao J., Yuan H., Tumaneng K., Li H. (2012). Regulation of the Hippo-YAP pathway by G-protein-coupled receptor signaling. Cell.

